# Cœsarian Operation after the Death of the Mother

**Published:** 1858-01

**Authors:** 


*Cœsarian Operation after the Death of the Mother.*—From a Bulletin of the *Académie de Médecine*, on the 17th November, found in the Gazette Hebdomadaire of the 20th of the same month, we learn that M. Velpeau reported to that body a successful Cœsarian operation performed twenty minutes after the the death of the mother. The mother had arrived at the full term of pregnancy. The operation was performed by M. Le-rey—Desbarres Surgeon of Hotel—Dieu of St. Denis. We hope to have soon a more extended report.

